# Cognitive Distortions and Alexithymia: A Cross-Sectional Study of Their Association

**DOI:** 10.3390/healthcare14142205

**Published:** 2026-07-21

**Authors:** Ibrahim Abdul Jaleel Yamani, Alean Al-Krenawi, Abd Elmureed Abd Elgaber Kaseem, Elwaleed Abdalla Farih Meiri, Emadeldin M. Elsokkary, Emad Mahgoub, Dhaval Vinodkumar Patel, Eid G. Abo Hamza

**Affiliations:** 1 Department of Psychology, College of Social Sciences, Imam Mohammad Ibn Saud Islamic University (IMSIU), Riyadh 13318, Saudi Arabia; iaymani@imamu.edu.sa (I.A.J.Y.); aamkasm@imamu.edu.sa (A.E.A.E.K.); emelsokkary@imamu.edu.sa (E.M.E.); 2 Resilience Research Centre, Dalhousie University, Halifax, NS B3H 4R2, Canada; 3 Department of Educational Psychology, Faculty of Education, Menoufia University, Shebin El-Kom, Menoufia 32511, Egypt; 4 Clinical Psychology Program, College of Public Health, Imam Abdulrahman Bin Faisal, University (IAU), Dammam 31441, Saudi Arabia; eamahgoub@iau.edu.sa; 5 Department of Psychology, Algoma University, Sault Ste. Marie, ON P6A 2G4, Canada; dhapatel@algomau.ca; 6 College of Arts, Humanities, and Social Sciences, University of Sharjah, Sharjah 27272, United Arab Emirates; 7 Department of Mental Health, Faculty of Education, Tanta University, Tanta 31527, Egypt

**Keywords:** cognitive distortions, alexithymia, emotional processing, gender differences, cognitive-behavioral interventions

## Abstract

**Background/Objectives**: The current study explored the relationship between cognitive distortions and alexithymia in 214 Saudi adults aged 20 to 45. **Methods**: Participants completed the Cognitive Distortions Scale and the Toronto Alexithymia Scale (TAS-20). **Results**: Results showed a significant positive correlation between cognitive distortions and alexithymia. Hierarchical regression analyses indicated that cognitive distortions were independently associated with overall alexithymia and difficulty identifying feelings after controlling for demographics, but not with difficulty describing feelings or externally oriented thinking. Gender moderation was non-significant across all outcomes, suggesting a similar cognitive–emotional pattern for both males and females. However, cognitive distortions partially accounted for the higher alexithymia scores observed in males, though this mediation finding should be interpreted cautiously given the unequal gender subgroup sizes. Culturally, these findings reflect norms in Arab societies, where emotional restraint—particularly among men—is often reinforced by values associated with honor and traditional masculinity. Such norms may inhibit emotional expression and contribute to higher levels of alexithymia. Additionally, collectivist orientations may foster cognitive styles that intersect with maladaptive cognitive distortions. **Conclusions**: These results highlight the need for culturally adapted cognitive-behavioral interventions that consider local beliefs about emotions and gradually enhance emotional awareness. Future research should examine the interplay of cultural, social, and religious factors with cognitive and emotional processes over time.

## 1. Introduction

Alexithymia is a complex trait of personality that is manifested by an inability to recognize, define, and interpret personal feelings. It is usually accompanied by an externally oriented cognitive style [1,2]. People with high levels of alexithymia can have difficulties with emotional awareness, interpersonal communication, and affect regulation, which exposes them to a higher risk of developing many psychological and somatic problems. These issues include depression, anxiety, addiction, and functional somatic disorders [3,4]. More recently, the Profile of Mental Functioning (M Axis) of the *Psychodynamic Diagnostic Manual, Third Edition* (PDM-3) has offered a complementary account of these same affective capacities, treating difficulties in identifying, modulating, and expressing emotion as falling along a continuum of functioning rather than as a fixed categorical deficit, a framing that anticipates the dimensional approach taken by the present study’s instruments [5].

During the last decades, the cognitive underpinnings of alexithymia have attracted increasing interest in research. Systematic biases and maladaptive thinking patterns also represent cognitive distortions that typically drive alexithymia owing to their adverse effect on emotional processing and self-perception [6,7]. These cognitive distortions and alexithymia have shown positive relationships with cognition and alexithymia in both clinical and non-clinical populations. However, the focus of past research was limited to students, psychosomatic symptoms, and general adults [8,9].

Recent studies have also highlighted the role of cognitive distortions in different behavioral and relational situations. For instance, Rana et al. [10] discovered that alexithymia and cognitive distortions also play a major role in the development of internet addiction among university students, with emotional intelligence as a mediating variable. In the same manner, Escarguel et al. [11] found a high level of alexithymia and cognitive distortion in people who perpetrated sexual crimes against children. This study has highlighted the importance of maladaptive thoughts that cause deviant behaviors. Zakeri and Rezaei [12] found that there is a negative correlation between alexithymia, dysfunctional attitudes, and marital intimacy in the context of intimate relationships. The researchers observed that the effect of cognitive distortions can disturb emotional expression and relationships.

Along with the above findings of research works across the globe, there is limited evidence of research in an Arab context. The majority of research targets specific groups of people, like those with sensory or learning difficulties [13]. Few studies have investigated alexithymia in normal adult groups, including university students [14,15]. A combination of evidence from within international and Arab settings indicates that cognitive distortions are critical factors in the emotional and behavioral manifestations of alexithymia, which can be considered as a factor requiring further study in various populations.

The existing literature on international topics of the last 20 years shows that the study of alexithymia, particularly in combination with cognitive distortions, automatic thoughts, irrational beliefs, and maladaptive schemas, is gaining interest. Initial clinical data also found that alexithymia and maladaptive automatic thoughts show significant positive correlations in patients with chronic headache [9]. These past studies suggest that alexithymia has a positive correlation with cognitive distortions, irrational beliefs, and cognitive failures. In turn, alexithymia could be used as a predictive value of dysfunctional thinking [7,10,16,17,18]. Taken together, these results highlight the multifaceted cognitive structure of alexithymia, which involves both a lack of emotional awareness and the systemic disruption of cognitive appraisal processes.

In Arab research, there has been a similar growth in studies on alexithymia in psychological and educational settings. However, most of the research has mainly concentrated on its prevalence and its relations with emotional, behavioral, and social factors such as academic procrastination, personality traits, social support, quality of life, and suicidal tendencies [19,20,21]. In addition, other studies have investigated alexithymia among clinical or special-needs groups [10,22,23]. Studies determined the effectiveness of counseling and therapeutic interventions in alleviating alexithymia [22]. Nevertheless, little focus has been given to the cognitive correlates of alexithymia, especially cognitive distortions. Research studies on non-clinical adult samples are very limited, with few exceptions [14]. This gap raises the importance of the current study for exploring the correlation between alexithymia and cognitive distortions in non-clinical adults in the Arab population. Consequently, the current research seeks to investigate the interrelationship between cognitive distortions and alexithymia and the cognitive mechanisms underlying alexithymia. Furthermore, it explores demographic disparities based on sex and education level, ultimately determining the extent to which cognitive distortions serve as predictors of alexithymia. By doing so, the research aims to inform the design of targeted clinical interventions, such as Cognitive-Behavioral Therapy (CBT) and emotional intelligence training, designed to mitigate dysfunctional schemas and enhance emotional processing.

### Arab Culture and Alexithymia

Cultural context is treated here as a potential moderator of the cognitive–emotional processes under study, rather than as a fixed, uniform property of Arab populations. This framing is supported by existing cross-cultural alexithymia research: the association between alexithymia and depression has been found to be stronger in collectivist cultural contexts than in individualist ones, a pattern attributed to culturally reinforced norms of emotional control rather than to any inherent difference in underlying emotional capacity [18]. Read in this way, the cultural factors discussed below are offered as candidate moderating mechanisms that may shape how cognitive distortions relate to alexithymia in this sample, rather than as a description of a single, homogeneous Arab psychology; meaningful variation across countries, generations, social classes, and urban and rural settings within the Arab world should be assumed unless demonstrated otherwise.

Collectivist orientations, which emphasize family cohesion, social harmony, and deference to hierarchy, have direct empirical support in Saudi samples: compared with American counterparts, Saudi respondents have shown a stronger tendency toward in-group-serving and out-group-derogating attributions, consistent with a more relational style of explaining social behavior [24]. To the extent that such orientations encourage individuals to subordinate the disclosure of personal distress to the maintenance of group harmony, they offer one plausible, testable mechanism by which externally oriented thinking, a core feature of alexithymia, could be reinforced through everyday social practice rather than through any deficit in emotional capacity per se; this remains a hypothesis to be tested directly rather than an established causal pathway.

Constructs of honor and traditional masculinity have similarly been linked to restricted emotional expression among men in Arab societies, with masculinity ideology described as promoting resilience and emotional suppression in ways that can elevate vulnerability to mental health difficulties when distress is not articulated [25,26]. If men’s socialization in this context provides less practice in naming and articulating internal states, rather than reflecting a genuine absence of emotion, this would be consistent with the present finding that male participants scored higher on difficulty identifying and describing feelings: a difference in trained expression rather than necessarily in underlying affective capacity. This distinction is also consistent with the modest size of the cognitive-distortions contribution to the gender gap reported in Section 5, which leaves most of that gap attributable to factors other than distorted cognition.

Religious and spiritual frameworks may offer a further, more speculative, candidate mechanism. Concepts such as patience (ṣabr) and reliance on God (tawakkul) are central to lived religious practice in Saudi and many broader Arab contexts and could, in principle, channel personal distress toward moral or spiritual interpretation rather than toward emotional labeling, while also supporting coping. To our knowledge, this possibility has not been directly tested in relation to alexithymia, and we present it here as a hypothesis for future empirical work rather than as an established finding; the same caution applies to suggestions that somatization, the expression of distress through physical symptoms, is differentially common in this cultural context, which was not directly assessed in the present study.

Taken together, these cultural factors are best treated as candidate moderators of the cognitive distortions–alexithymia association documented in this study. Some, such as collectivist attribution style and masculinity-linked emotional restraint, have direct empirical support in Arab and Saudi samples [24,25], while others, such as religious coping framings and somatization, remain plausible but currently untested in this literature and should not be presented with more confidence than the evidence allows. Clinically, this reframing is important: instruments such as the TAS-20 may partly reflect culturally normative communication styles in addition to individual differences, and interventions such as CBT may need to build emotional vocabulary and incorporate family and religious context as a precondition for engagement, rather than treating emotional expression as a culturally neutral skill to be taught in isolation. None of this implies that Arab culture inherently produces alexithymia; rather, cultural norms appear to shape how, and how readily, emotional experience is identified, expressed, and reported, which is precisely the moderating role these factors are proposed to play here.

## 2. Theoretical Background

### 2.1. Alexithymia

The term alexithymia was first coined by Sifneos [27] as a result of his clinical observations of psychosomatic patients with observed significant losses in the ability to identify and verbalize their emotions. Impairments go beyond internal processing and can lead to serious problems in the context of interpersonal communication and social-affective interaction [7]. Alexithymia is defined by Taylor et al. [1] and Kinnaird et al. [2] as a personality trait characterized by diminished emotional and affective processing and an externally focused cognitive orientation. On the same note, Ameri et al. [28] also conceptualized alexithymia as the defensive response to anxiety, internal conflict, and psychological trauma, which leads to maladaptive failure to control or manage emotions.

Based on the aforementioned perspectives, alexithymia is defined in this study as a personality trait marked by difficulties in perceiving and regulating emotions, alongside challenges in recognizing and interpreting the emotions of others.

This definition can be further anchored within the *Psychodynamic Diagnostic Manual, Third Edition* (PDM-3) [5], whose Profile of Mental Functioning (M Axis) offers a clinically rich, dimensionally nuanced account of affective capacities, including the capacity to identify, modulate, and express emotion, that directly intersects with alexithymia as operationalized here. Unlike a purely categorical reading of the construct, the M Axis treats emotional-processing difficulties not as a fixed absence but as a continuum of functioning that varies in degree across individuals, a framing that is considerably more consistent with the continuous scoring of the TAS-20 used in this study than a categorical present/absent conception of alexithymia would be. This dimensional perspective is revisited in the Discussion when interpreting why cognitive distortions in this sample were associated with one facet of alexithymia and not others.

### 2.2. Cognitive Distortions

Cognitive distortions have also been theorized as the mediating factor between latent cognitive schemas and automatic thoughts [6,29,30]. In accordance with Beck’s cognitive theory [29], these maladaptive schemas represent irrational beliefs or assumptions that actively shape an individual’s perceptions of both the self and their social environment. Cognitive distortions represent cognitive patterns, which act as the hidden framework by which social information is processed and perceived [31]. Moreover, Clark et al. [32], Jager-Hyman et al. [33], and Yurica and DiTomasso [34] conceptualize cognitive distortions as logical fallacies that induce a systematic negative bias within an individual’s information-processing framework. Likewise, Dozois and Beck [35] outlined them as automatic prejudices and counterproductive thought patterns that induce psychological suffering [36].

This paper, therefore, posits that cognitive distortions are biased thought patterns that intensify during periods of psychological stress, leading to persistently negative views of the self and the surrounding world. Thus, the operational definition of cognitive distortions hereafter is “*A group of negative thinking patterns used by an individual in social spheres, i.e., relationships with friends, family, and colleagues, and measured by the overall score of the Cognitive Distortions Scale used in the study*”.

### 2.3. Theoretical Evolution: From Clinical Description to Attention-Appraisal Mechanisms

The psychological construct of alexithymia has gone through a paradigm shift since its introduction by the psychiatrist Peter Sifneos in 1973 [27]. It was originally identified in patients with psychosomatic conditions but initially conceived as an absence of words to describe emotions (a-lexis-thymos). It was expressed through an impoverished imagination and utilitarian approach to thinking [4,27]. Moreover, modern theoretical models have gone beyond these descriptions and are process-oriented in their interpretations. In a historical work, Preece et al. [37] came up with the Attention-Appraisal Model, which incorporates alexithymia into the wider context of the Extended Process Model of Emotion Regulation. According to this model, alexithymia is not an issue of language deficiency, but a systemic failure in two key processes: (1) the attention process, where people cannot orient their attention to internal states because of externally focused thinking (EOT), and (2) they cannot effectively label the states as certain specific emotions because of an inability to do so (Difficulty Identifying Feelings (DIF) and an ability to do so (Difficulty Describing Feelings (DDF) [37,38].

The view is further supported by the development of a valence-specific model. Recent findings utilizing the Perth Alexithymia Questionnaire (PAQ) in 2023 underscore that alexithymia impairments are transdiagnostic across affective domains, though individuals typically demonstrate more pronounced difficulties in processing negative affect [39]. The shift from a categorical to a dimensional paradigm has enabled researchers to conceptualize alexithymia as a stable personality trait distributed along a continuum. In this framework, clinically significant levels are estimated to persist in 5% to 19% of the general population [40].

### 2.4. Transdiagnostic Pathophysiology: Meta-Analytic Foundations

The clinical implications of alexithymia lie in the fact that alexithymia is a strong transdiagnostic risk factor of various psychiatric diagnoses, such as depression, anxiety, schizophrenia, and post-traumatic stress disorder (PTSD) [38]. Liu et al. [18] found a significant positive correlation between total alexithymia and depression severity (r = 0.457–0.459) in a meta-analysis of 35 studies. They found that DIF is the most robust predictor of clinical symptoms, whereas EOT can often be less significant or insignificant in relation to affective disorders [18,41]. According to this, the appraisal deficit is more central in psychopathology than the attention deficit. These relationships are highly moderated by culture. Liu et al. [18] noted that the relationship between alexithymia and depression is more correlated in Eastern collectivist cultures than in Western individualistic cultures. This is postulated to be due to cultural beliefs such as the Confucian doctrine of the Mean, which advocates emotional control and suppression and thus increases internal issues of affective confusion [18]. In addition to this, Reyno et al. [42] demonstrated a robust association between alexithymia and dissociation, characterizing it as a functional impairment in the process of integrating and labeling internal emotional experiences. This results in primitive and avoidant defenses, including projection and denial, which makes it impossible to incorporate affect into conscious memory [42].

Behavioral addictions can also be classified as transdiagnostic. According to Rana et al. [10], alexithymia and cognitive distortions play a critical role in internet addiction in university students. The same relationships were observed in the case of pathological gambling when accusers utilize external stimulation to balance internal emotional ambiguity [7,43]. In addition, Hemming et al. [41] found a huge effect size (r = 0.54) between alexithymia and suicide ideation, which reinforces the essential role of the construct in clinical risk assessment.

### 2.5. The Neurobiological Architecture of the Alexithymic Brain

Neuroimaging studies also provide a biological explanation of cognitive-affective failures observed in alexithymia. In a meta-analysis of fMRI research, van der Velde et al. [44] discovered that high alexithymia is linked to reduced neural reactions to emotional stimuli in certain areas. These areas are responsible for emotion recognition and interoceptive insight, specifically the anterior insula and the amygdala. The insula serves as a central interception zone that regulates internal sensing. A decrease in anterior insula activation when processing emotions indicates a lack of so-called emotional granularity, or the possibility to distinguish between various affective states in finer detail [44].

On the contrary, people with high alexithymia levels show more activity in the posterior insula and the somatosensory parts of the brain during primitive emotional responses, such as pain [4,44]. According to Kano and Fukudo [4], it is an increase in the level of awareness of raw physical arousal without the cognitive ability to identify it as an emotion, which is a process that is often referred to as somatization. These neural disruptions have also been supported by EEG findings. In a mechanistic review, Di Benedetto et al. [45] found that alexithymic participants had right-hemisphere dominance in EEG power and connectivity, especially in the theta and alpha bands. This implies an impairment in the interhemispheric transfer that does not allow the symbolic elaboration of feelings to take place, and instead retains emotional experiences in their pre-verbal form [38,45].

Advanced imaging has also revealed specific network dysconnectivity. Alexithymia is strongly associated with dysconnectivity within the semantic network and altered functional connectivity between monoaminergic brainstem regions (such as the dorsal raphe and locus coeruleus) and cortical areas crucial for social cognition, including the medial prefrontal cortex (mPFC) and the inferior parietal lobule [46]. These alterations suggest that alexithymia involves a less specialized and less efficient neural processing of socio-emotional information [46].

### 2.6. Cognitive Distortions and Maladaptive Schemas: Mechanistic Pathways

The development of alexithymia is increasingly tied to systematic cognitive biases and dysfunctional internal templates. Cognitive distortions such as catastrophizing, all-or-nothing thinking, and emotional reasoning function as the engine that fuels affective failure [6,29]. Rnic et al. [6] posited a reciprocal association between cognitive distortions and emotional dysregulation, wherein biased cognitions intensify affective instability. This dysregulation serves to solidify and reinforce distorted thinking patterns, which establish a self-perpetuating maladaptive cycle.

Moreover, these distortions arise from early maladaptive schemas (EMSs). Pilkington et al. [47], in a systematic meta-analysis of the existing body of evidence on EMS areas, have identified nearly 18 domains. The study found that all identified domains were significantly involved in alexithymia, especially those concerning unmet needs of attachment and autonomy. Emotional inhibition (r = 0.50) and social isolation (r = 0.44) demonstrate the most significant associations with alexithymic characteristics [47]. Saritas-Atalar and Gencoz [48] also indicated that in a path analysis, EMSs mediate the correlation between early childhood trauma exposure and adulthood levels of alexithymia. This developmental trajectory implies that people in invalidating environments are taught that it is unsafe or ineffective to express emotional experiences and develop an ability deficit in schematically forming emotional experiences [48].

The most extreme result of such cognitive disturbances is cognitive failure. Using a study of adolescents, Liu et al. [18] discovered that alexithymia serves as a considerable portion of the ratio of prediction of cognitive failure, i.e., errors in simple daily activities such as memory lapses or attention management (r = 0.65). This means that the inability to process emotions effectively occupies huge resources of cognitive power, and fewer resources are available to carry out daily tasks and navigate the social environment [18].

### 2.7. Conceptual Framework

The current research encompasses a combined conceptual approach that incorporates the use of four theoretical perspectives that complement each other to demonstrate how cognitive distortions are predictors of alexithymia. Cognitive Appraisal Theory [49] posits that emotions are a result of subjective cognitive evaluation of events and stimuli. When cognitive distortions systematically bias these appraisals, for instance, through catastrophizing or all-or-nothing thinking, the individual’s capacity to accurately identify and label internal emotional states becomes impaired. The theory of schema [29,50] suggests that early maladaptive schemas form highly inflexible catalogs of thought that determine the manner in which emotional information is taken up by people. Such schemas continue the distorted thinking patterns and interception of accurate emotional labeling and thus promote alexithymic characteristics. According to Emotion Regulation Theory [51], to be an effective emotional regulator, the individual must be able to properly recognize affective states during the appraisal phase. This is a disrupted stage that is directly interfered with by cognitive distortions. Since this appraisal process is undermined, the individual finds it difficult to control their emotions. This subsequently culminates in the typical deficit of alexithymia. Lastly, the Attention-Appraisal Model [37] is a direct mechanistic explanation, which places alexithymia in the context of attention and appraisal phases of emotional processing and aligns alexithymia precisely with three dimensions of alexithymia, i.e., DIF, DDF, and EOT.

**Figure 1:**
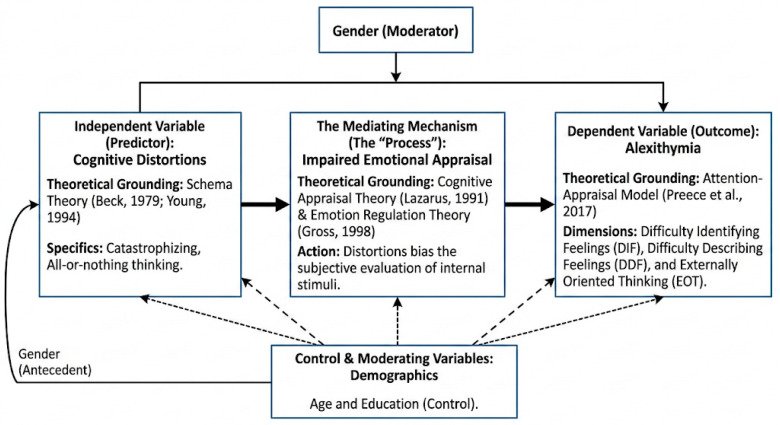
Conceptual model of cognitive distortions, impaired emotional appraisal, and alexithymia with demographics as moderators [37,49,50,51,52].

These theoretical views unite in the conceptual model in Figure 1. Cognitive distortions undermine the proper evaluation of internal emotional states. This results in emotional processing losses, which subsequently translate to alexithymia. This is the highest in the dimension of difficulty identifying feelings (DIF). There are demographic variables (gender, age, and education) that are controlled in the analysis. Gender is also a possible moderator of the alexithymia linkage with distortion, as well as a primary antecedent variable in the mediation hypothesis. The entire conceptual framework is provided in Figure 1.

### 2.8. Mentalized Affectivity: A Complementary Construct

A construct that has not previously been engaged with in this literature, but that shares important conceptual overlap with alexithymia, is mentalized affectivity [53,54]. Mentalized affectivity refers to the reflective capacity to identify, process (modulate and differentiate), and express emotional states in light of one’s autobiographical and interpersonal history. It is not simply the inverse of alexithymia: where alexithymia is defined by deficit, mentalized affectivity foregrounds the reflective and meaning-making dimensions of emotional life, and its three components map onto, without being identical to, the TAS-20 dimensions used here, identifying emotions overlapping with difficulty identifying feelings (DIF), processing and expressing emotions overlapping with difficulty describing feelings (DDF), and externally oriented thinking (EOT). Validated measures of the construct, including the Mentalized Affectivity Scale and its briefer form, the Brief Mentalized Affectivity Scale, have since been extended to Italian and adolescent samples [54,55,56,57], demonstrating that the construct travels across cultural and developmental contexts, though no Arabic-language validation currently exists.

Conceptualizing the present study through this lens generates a testable possibility, returned to in the Discussion, that cognitive distortions disrupt the reflective, identification-level component of mentalized affectivity specifically, while leaving its more relational and expressive components governed primarily by other factors, such as the cultural norms around emotional disclosure discussed above. Administering a mentalized affectivity measure alongside the TAS-20 and Cognitive Distortions Scale in future research would allow this possibility, and the broader conceptual overlap and divergence between the two constructs, to be tested directly rather than inferred post hoc.

## 3. Materials and Methods

### 3.1. Research Design

This research used a descriptive correlational research design to examine the correlation between cognitive distortions and alexithymia in adults. It also validated the power of forecasting cognitive distortions in interpreting differences in the level of alexithymia. The correlational approach was deemed appropriate because it allows investigation of associations between psychological variables without experimental manipulation, relying on standardized and psychometrically validated instruments.

### 3.2. Participants

Before determining the sample size, it is essential to define the study setting and the target population. Prior identification of the target population will help the researcher to choose a fair and representative sample that is representative of the entire population [58]. Administrative staff, teachers, and psychologists in various educational institutions in Riyadh were sampled to recruit 214 adults (152 males and 62 females) aged 20 to 45 years (mean = 30.9, SD = 5.2). Table 1 provides the demographic attributes of the sample. Each member was asked to fill in two pencil-and-paper assessments in counterbalanced sequence to eliminate the possibility of sequence effects: the Arabic version of the Cognitive Distortion Scale and the Toronto Alexithymia Scale (TAS-20). Inclusion criteria were: (a) Arabic-speaking adults aged 18–60 currently employed at an educational institution in Riyadh; and (b) willingness to provide written informed consent. Exclusion criteria were: (a) a self-reported current diagnosis of a major psychiatric disorder (e.g., schizophrenia, bipolar disorder); (b) ongoing psychotropic medication use that could confound self-report validity; and (c) incomplete questionnaire responses (less than 90% item completion). Participants were approached through a convenience sampling strategy: the first author circulated study information via departmental coordinators at their institution and affiliated institutions. Participation was voluntary, anonymous, and yielded no academic or employment benefit to discourage socially desirable responses. The gender imbalance in the final sample (71% male) reflects the staffing composition of the institutions approached rather than deliberate sampling choices, and its interpretive implications are addressed in Section 6.

### 3.3. Instruments

#### 3.3.1. Cognitive Distortions Scale (CDS)

The Cognitive Distortions Scale (adapted by the researcher into Arabic) was initially designed by Covin, Dozois, Ogniewicz, and Seeds [59] who evaluated ten forms of cognitive distortions, i.e., hostile attribution, catastrophizing, all-or-nothing thinking, emotional reasoning, labeling, overgeneralization, personalization, should statements, mental filtering, and minimization of the positive. The scale has 20 items in two areas (a) performance and achievement in academic or occupational situations, and (b) social relations with family and friends. The answers are graded using a 7-point Likert scale (1 = never, 7 = always) and the overall marks total to between 20 and 140. The greater the scores, the higher the cognitive distortions. The original scale had very good psychometrics, such as a Cronbach’s α of 0.90 and a test–retest of 0.88 over a two-week interval. A unidimensional structure was supported by factor analysis. Further validation was done by Ozdel et al. [60], where Cronbach alpha coefficients ranged between 0.87 and 0.94, indicating that there was high internal consistency and reliability. The Arabic version of the Cognitive Distortions Scale (CDS) was prepared using translation procedures. Two bilingual psychologists separately translated the English version into Arabic, and the translations were reconciled into a single version. Independent bilingual translators later back-translated this version into English, with any discrepancies resolved through discussion, to ensure it was semantically and conceptually equivalent to the original instrument. A review of the Arabic version was completed by a panel of 10 psychology experts to ensure the validity of the language, culture, and content. Expert consensus was between 80% and 100%, with only minor wording changes made. The Arabic CDS in the present sample was found to have good internal consistency (Cronbach’s α = 0.84) and excellent split-half reliability (Spearman–Brown = 0.94).

#### 3.3.2. Toronto Alexithymia Scale (TAS-20)

The Toronto Alexithymia Scale (TAS-20), which was designed by Bagby et al. [61] and translated into Arabic by Kafafi and Al-Dawash [62], assesses three dimensions, namely difficulty in identifying feelings, difficulty in describing feelings, and externally oriented thinking. The scale has 20 questions, which are rated on a 5-point Likert scale (1 = strongly disagree, 5 = strongly agree), and scores are based on reverse scoring on questions with a negative word. The total scores range between 20 and 100; a higher score suggests a greater level of alexithymia. The original TAS-20 had good psychometric qualities (a = 0.80), whereas the Arabic version had high reliability (test–retest r = 0.89) and good validity, with an important negative relationship (r = −0.78) with the Meta-Mood Scale.

### 3.4. Procedure

The data and the Diploma of Education program included for study were collected during sessions of 10–30 students in a lecture, led by colleagues of the faculty working at the Department of Psychology at Imam Mohammad Ibn Saud Islamic University (IMSIU). Sessions were about 30–50 min long. Instruments were administered individually to faculty members and administrative staff from various colleges at the university, with each session taking approximately 30–40 min.

Data collection occurred between September and November 2025, with logistical support from colleagues in the Department of Psychology, College of Social Sciences, Imam Mohammad Ibn Saud Islamic University (IMSIU).

### 3.5. Statistical Data Analysis

Following data collection, the dataset was analyzed using the Statistical Package for the Social Sciences (SPSS, Version 26). Percentages and frequency distributions were initially computed. After data verification, mean scores, standard deviations, and skewness values for the scales used in the present study were calculated. Additionally, Pearson’s correlation coefficient, independent-samples *t*-tests, and one-way ANOVA were employed to examine group differences. Two-block hierarchical multiple regression analyses were conducted to examine the unique predictive contribution of cognitive distortions beyond demographic variables (gender, age, and education). Moderation analysis tested whether gender moderated the cognitive distortions–alexithymia relationship using interaction terms. Mediation analysis was performed using PROCESS macro v4.0 in SPSS [63] with 5000 bootstrap resamples to estimate indirect effects with 95% confidence intervals. Effect sizes were reported using Cohen’s d, η^2^, and Cohen’s f^2^, where applicable.

## 4. Results

### 4.1. The Relationship Between Cognitive Distortions and the Dimensions of Alexithymia

Correlations were calculated between the participants’ scores on cognitive distortions and alexithymia. The participants’ scores are presented in Table 2.

Findings revealed that cognitive distortions were positively and significantly associated with all alexithymia dimensions. Specifically, cognitive distortions showed a moderate positive correlation with difficulty identifying feelings (*r* = 0.29, *p* < 0.01) and a weak but significant correlation with difficulty describing feelings (*r* = 0.19, *p* < 0.01). A weaker yet statistically significant relationship was found with externally oriented thinking (*r* = 0.14, *p* < 0.05). Also, cognitive distortions showed a positive relationship with the total alexithymia score (r = 0.28, *p* < 0.01). In the context of interrelationships between alexithymia dimensions, a strong positive relationship occurred. Difficulty identifying feelings was closely related to difficulty describing feelings (r = 0.62, *p* < 0.01), and very strongly related to the overall score regarding alexithymia (r = 0.85, *p* < 0.01). In the same way, a very strong relationship was seen between difficulty describing feelings and the total score (r = 0.84, *p* < 0.01). Moderately positive correlations were also observed between externally oriented thinking and difficulty describing feelings (r = 0.35, *p* < 0.01) and the overall alexithymia score (r = 0.60, *p* < 0.01).

### 4.2. Gender Differences in Cognitive Distortions and Alexithymia

An independent samples *t*-test was applied to test gender differences in dimensions of cognitive distortions and alexithymia (see Table 3).

As shown in Table 3, statistically significant differences emerged across all variables, with males consistently reporting higher scores than females. Regarding externally oriented thinking, males (*M* = 15.69, *SD* = 3.94) again scored significantly higher than females (*M* = 11.85, *SD* = 3.84), *t*(212) = 6.50, *p* < 0.001, with a large effect (*d* = 0.98), suggesting a stronger tendency among males toward externally focused, concrete thinking styles. Finally, the total alexithymia score showed the largest gender disparity. Males (*M* = 61.12, *SD* = 9.83) scored significantly higher than females (*M* = 49.06, *SD* = 9.66), *t*(212) = 8.18, *p* < 0.001. The effect size was very large (*d* = 1.23), indicating a pronounced overall difference in emotional awareness and processing.

### 4.3. Differences According to Educational Level

In order to test the variation in alexithymia and cognitive distortions in relation to education level, the sample was divided into three groups of respondents, i.e., those with a Bachelor’s, Master’s/Diploma, and PhD. To determine statistically significant differences between these groups, one-way analysis of variance (ANOVA) was performed. Table 4 displays the results.

ANOVA analysis revealed no statistically significant differences in cognitive distortions across educational groups, *F*(2, 211) = 0.25, *p* = 0.776. The effect size was negligible (η^2^ = 0.002), indicating that educational level accounted for less than 1% of the variance in cognitive distortions.

Similarly, differences in the total alexithymia score across educational levels did not reach statistical significance (*F*(2, 211) = 2.15, *p* = 0.119). Although the between-group variance was slightly higher than that observed for cognitive distortions, the effect size remained small (η^2^ = 0.020), suggesting that educational attainment explains only a minimal proportion of variance in alexithymic traits. In general, the results reveal that educational level does not seem to be a significant variable that may affect maladaptive cognitive patterns or problems with emotional awareness in the study sample.

### 4.4. Hierarchical Regression: Predicting Alexithymia from Cognitive Distortions

Analysis to investigate the distinct predictive value of cognitive distortions in alexithymia over demographic variables involved a succession of two-block hierarchical multiple regression equations on each of the four outcome variables, namely total alexithymia score, difficulty identifying feelings (DIF), difficulty describing feelings (DDF), and externally oriented thinking (EOT). The demographic control variables were input in Block 1 (gender, age-centered, Master’s vs. Bachelor’s, PhD vs. Bachelor’s). The cognitive distortions score was included in Block 2. Cohen’s f^2^ was used to quantify effect sizes. The findings are provided in Table 5 and Table 6.

As shown in Table 5, Block 1 demographic predictors collectively explained 33.5% of the variance in total alexithymia (R^2^ = 0.335, adj-R^2^ = 0.323, F(4, 209) = 26.37, *p* < 0.001, f^2^ = 0.504), indicating a large effect. Gender emerged as the dominant predictor (B = 13.19, SE = 1.47, β = 0.536, *p* < 0.001), with males scoring substantially higher on alexithymia than females. Age was a significant negative predictor (B = −0.40, SE = 0.13, β = −0.186, *p* = 0.002), suggesting slightly lower alexithymia with older age. Having a PhD (vs. Bachelor’s) was also negatively associated with alexithymia (B = −6.73, SE = 1.72, β = −0.233, *p* < 0.001), while the Master’s/Diploma coefficient was not significant (*p* = 0.122).

The addition of cognitive distortions in Block 2 produced a statistically significant increment in explained variance (ΔR^2^ = 0.019, ΔF(1, 208) = 6.06, *p* = 0.015), bringing the total R^2^ to 0.354. The standardized coefficient for cognitive distortions was β = 0.143 (B = 0.077, SE = 0.031, 95% CI [0.015, 0.139], *t* = 2.46, *p* = 0.015), indicating that higher levels of cognitive distortions predicted higher alexithymia even after controlling for demographic variables, albeit with a small incremental effect (f^2^ = 0.029).

As shown in Table 6, cognitive distortions significantly predicted difficulty identifying feelings (DIF) after controlling for demographics (β = 0.177, ΔR^2^ = 0.029, ΔF(1, 208) = 8.34, *p* = 0.004), with a 95% confidence interval for B of [0.016, 0.087]. In contrast, cognitive distortions did not significantly predict difficulty describing feelings (DDF; β = 0.043, *p* = 0.503) or externally oriented thinking (EOT; β = 0.079, *p* = 0.197) once demographics were controlled. These results indicate that the relationship between cognitive distortions and alexithymia is confined to the emotional recognition aspect of alexithymia and not to descriptive or externally focused facets of alexithymia.

### 4.5. Moderation Analysis: Gender as a Moderator

To examine whether gender moderates the relationship between cognitive distortions and alexithymia, a series of moderation analyses were conducted following the procedures outlined by Hayes [63]. For each outcome variable, a Gender × Cognitive Distortions interaction term was entered in a third block (Block 3), after controlling for demographics (Block 1) and the main effect of cognitive distortions (Block 2). Cognitive distortions were mean-centered before computing the interaction term.

The interaction term was non-significant across all outcomes: total alexithymia (ΔR^2^ < 0.001, ΔF(1, 210) = 0.001, *p* = 0.973), difficulty identifying feelings (ΔR^2^ = 0.006, ΔF(1, 210) = 1.55, *p* = 0.215), difficulty describing feelings (ΔR^2^ < 0.001, *p* = 0.891), and externally oriented thinking (ΔR^2^ = 0.009, ΔF(1, 210) = 2.38, *p* = 0.125). These findings suggest that there is no significant gender difference in the relationship between cognitive distortions and alexithymia. That is, cognitive distortions were predicted as a similar predictor of alexithymia between both gender groups, which indicates that there is no difference in the cognitive pathway to alexithymia between genders.

### 4.6. Mediation Analysis: Cognitive Distortions as a Mediator of the Gender–Alexithymia Link

Given the significant gender gap in both cognitive distortions and alexithymia scores, a mediation analysis was conducted to test whether cognitive distortions partially account for the gender–alexithymia association. Using PROCESS macro v4.0 in SPSS with 5000 bootstrap resamples, the indirect effect of gender (coded 1 = male, 2 = female) on each alexithymia outcome via cognitive distortions was estimated, controlling for age and educational level.

Findings showed that gender had a serious indirect influence on total alexithymia via distortion of the mind (ab = 0.903, SE = 0.439, Boot 95% CI [0.082, 1.955]), comprising 6.4% of the overall impact of gender on alexithymia via distortion of the mind. Likewise, there was an important indirect pathway of the challenge of discovering feelings (ab = 0.602, SE = 0.264, Boot 95% CI [0.132, 1.264]) that explained 9.2% of the total gender effect on DIF.

On the contrary, the indirect effects through difficulty explaining feelings (ab = 0.106, SE = 0.166, Boot 95% CI [−0.221, 0.432) and externally oriented thinking (ab = 0.226, SE = 0.192, Boot 95% CI [−0.126, 0.611) were not statistically significant, since the confidence intervals covered zero. Collectively, these findings suggest that cognitive distortions serve as a partial mediator between gender and alexithymia specifically through the emotional recognition pathway (DIF), but not through verbal or external thinking components. These mediation findings should, however, be interpreted with caution: the female subsample (*n* = 62) is substantially smaller than the male subsample (*n* = 152), and the bootstrap confidence intervals for the indirect effects are correspondingly wide. The partial mediation through DIF is a preliminary finding that requires replication in a gender-balanced sample before stronger conclusions can be drawn about the mechanism linking gender to emotional recognition difficulties in this population.

## 5. Discussion

The present study examined the concurrent association between cognitive distortions and alexithymia in a non-clinical Saudi adult sample. The most theoretically significant finding is not the overall positive correlation, which the existing literature would lead one to expect [6,7], but the differential pattern across the three TAS−20 subscales: cognitive distortions were independently associated with difficulty identifying feelings (DIF) after controlling for demographics, but not with difficulty describing feelings (DDF) or externally oriented thinking (EOT). This subscale specificity invites an interpretive question that the data alone cannot settle but that the present theoretical framing helps to address: why should cognitive distortions bear a relationship specifically to the emotional recognition component of alexithymia?

Within the Attention-Appraisal Model, DIF is positioned as a failure at the appraisal phase of emotional processing—the inability to label internal states correctly once attention has been oriented toward them [37]. Cognitive distortions, which by definition systematically bias the appraisal of self-relevant information, are precisely the kind of mechanism one would expect to disrupt this labeling step specifically, rather than the subsequent communication (DDF) or attentional orientation (EOT) steps. The finding that DIF, but not DDF or EOT, is independently associated with cognitive distortions is therefore theoretically coherent and consistent with the Attention-Appraisal account. This interpretation is also compatible with the PDM−3 M Axis framing discussed in Section 2.1: if emotional-processing capacity is dimensional rather than categorical, then cognitive distortions may represent one of several inputs that push functioning toward the lower end of the identifying-emotions dimension while leaving other affective capacities relatively intact [5]. The same logic informs the mentalized affectivity framework introduced in Section 2.8: distorted cognition appears to specifically disrupt the identification component of mentalized affectivity, while the processing and expressing components may be governed primarily by other factors, consistent with the conclusion that DDF and EOT in this sample were more strongly patterned by socialization-related variables, particularly gender, than by cognitive distortions [53,54].

A second major finding concerns the relative magnitude of effects. Demographic variables, with gender as the dominant contributor (β = 0.536, Block 1 f^2^ = 0.504), collectively accounted for a much larger proportion of variance in total alexithymia than did cognitive distortions incrementally (ΔR^2^ = 0.019, f^2^ = 0.029). Rather than treating this as a negative result, the appropriate interpretation is that socialization processes, and gender socialization in particular, appear to be more fundamental drivers of alexithymia in this sample than cognitive distortions alone. This does not undermine the study’s value; it reframes the contribution of cognitive distortions as one element within a broader, socially embedded account of why certain individuals, and certain groups, develop difficulties with emotional identification. In the specific context of Arab gender norms, males in this sample may have been socialized through a combination of honor-related emotional restraint and restricted emotional vocabulary development toward patterns of thought and expression that produce higher scores on both cognitive distortion measures and alexithymia scales, with the former partially accounting for the latter in the DIF dimension, but with most of the gender effect on DDF and EOT traveling through pathways that are not captured by cognitive distortion scores.

The moderation finding, that the cognitive distortions–alexithymia association does not differ significantly by gender (all interaction ΔR^2^ < 0.01), is also theoretically informative. It suggests that the cognitive pathway to emotional recognition difficulties is qualitatively similar for men and women, even though the absolute level of alexithymia differs substantially between groups. The mediation analysis adds nuance: cognitive distortions partially account for the gender difference in DIF (9.2% of the total gender effect), and partially for the total alexithymia score (6.4%), but do not explain the gender differences in DDF or EOT. Together, these findings are consistent with the interpretation that cognitive distortions operate as a shared cognitive mechanism for impaired emotional recognition across genders, while the overall pattern of gender differences in alexithymia involves mechanisms well beyond distorted cognition, including culturally shaped socialization experiences that differ substantially between males and females in this context.

A developmental framing is important for contextualizing these findings, and its absence from the earlier version of this manuscript represented a significant gap. Both alexithymia and cognitive distortions have well-documented developmental antecedents: early invalidating caregiving environments, limited modeling of emotional language within the family, and the consolidation of maladaptive cognitive schemas during adolescence have all been linked to adult alexithymia and to the early maladaptive schemas that underpin cognitive distortions in adulthood [47,48]. Path-analytic evidence indicates that early maladaptive schemas mediate the relationship between childhood trauma exposure and adult alexithymia [48], and that attachment-related schema domains, particularly emotional inhibition (r = 0.50) and social isolation (r = 0.44), are among the strongest correlates of alexithymic characteristics [47]. The present cross-sectional design cannot speak to these developmental antecedents directly, but it positions the observed cognitive–emotional associations within a plausible developmental chain: individuals raised in environments that discouraged emotional disclosure or failed to model emotional identification may have developed both greater cognitive distortions and more difficulty identifying feelings as a consequence, with the correlational association observed here reflecting shared developmental origins rather than a direct cross-sectional influence of one construct on the other. This has direct implications for intervention timing: if the cognitive–emotional patterns observed here consolidate during adolescence, early identification and psychoeducational programs that build emotional vocabulary alongside schema-focused work may be more effective than adult-stage interventions alone [64].

The clinical implications of these findings impact the design of intervention programs in this cultural context. Because the cognitive distortions–DIF association was replicated in both male and female participants, CBT-based cognitive restructuring techniques could plausibly be used with both groups to target emotional recognition difficulties, without requiring gender-differentiated therapeutic protocols at the level of the core cognitive technique. However, because most of the gender-related alexithymia variance, including the entirety of the DDF and EOT gender gap, remains unexplained by cognitive distortions, clinicians working with male participants in Arab contexts should anticipate that restructuring distorted cognitions alone will be insufficient to fully address alexithymia. Complementary approaches that build emotional vocabulary and gradually scaffold emotional articulation, adapted to honor-related norms around expressing personal vulnerability and conducted with attention to family system dynamics, will likely be needed as adjuncts [5]. The absence of a validated measure of psychological distress in this study means it is not possible to determine whether the alexithymia levels observed here were elevated relative to normative expectations or whether they co-occurred with subclinical symptomatology; this contextual information is important for grading the urgency of any intervention and should be included in future work. In practical terms, culturally adapted CBT for this population might include: (a) emotion-labeling exercises in which clients learn to name internal states using an Arabic emotional vocabulary list built collaboratively with the therapist, beginning with physical sensations before progressing to psychological labels, to minimize shame around emotional disclosure; (b) thought-record work framed around occupational and family roles (e.g., “as a father/provider, what am I telling myself in this situation?”) rather than individualistic self-referential framing, to align with collectivist self-construal; (c) psychoeducation about the distinction between emotional awareness and emotional expression, clarifying that developing an inner emotional vocabulary does not require breaking norms of public restraint; and (d) where possible, involvement of key family members in psychoeducational sessions, given the centrality of family dynamics to male emotional socialization in this context. These adaptations should be piloted and evaluated in randomized or quasi-experimental designs before being recommended as evidence-based practice for this population.

In terms of convergence with the existing literature, the present findings are broadly consistent with research linking cognitive distortions and related constructs such as irrational beliefs and automatic thoughts to alexithymia in other non-clinical and clinical populations [7,16,17], and align specifically with the meta-analytic finding that DIF is the most robust subscale correlate of cognitive and clinical variables, whereas EOT tends to show weaker or non-significant associations with psychopathological predictors [18,41]. The present results extend this pattern to the Saudi non-clinical context and demonstrate that it holds after controlling for gender, which had not been consistently addressed in prior work. A point of divergence from some earlier accounts is the size of the cognitive distortions contribution relative to gender: the present data suggest that in this sample, socialization-related factors indexed by gender represent a far larger source of alexithymia variance than distorted cognition, which should prompt a more cautious framing of cognitive distortions as one contributing factor among several, rather than as a primary driver of alexithymia in Arab adult populations.

Several directions follow for future research. Longitudinal designs are needed to establish whether the cognitive distortions–DIF association is directional, bidirectional, or driven by shared antecedents; the cross-sectional data presented here are equally consistent with all three possibilities, and the present study’s title and framing have been revised accordingly to avoid implying directionality that the design cannot support. Future work should include at least one validated measure of general psychological functioning or symptom severity alongside the two core scales used here, so that the clinical significance of observed alexithymia levels can be contextualized against normative expectations. Administering a validated measure of mentalized affectivity alongside the TAS−20 and CDS would allow the theoretical overlap proposed in Section 2.8 to be tested directly. Recruiting from more diverse samples across Arab countries, regions, generations, and socioeconomic strata would address the representativeness limitation inherent in a single-university Riyadh sample, and power analyses specifically factoring in gender subgroup sizes should guide future sampling strategies given the interpretive constraints imposed by the unequal gender distribution in the present study.

## 6. Conclusions, Limitations, and Future Research

The present study found that cognitive distortions were concurrently associated with difficulty identifying feelings in a non-clinical Saudi adult sample after controlling for demographic variables, though the association did not extend to difficulty describing feelings or externally oriented thinking. Given the cross-sectional design, these results are best understood as establishing that the two constructs co-vary, not as evidence that one causes the other; directionality remains an empirical question for future longitudinal work. The most substantive finding, in terms of explained variance, is the large gender difference in alexithymia across all subscales, which points toward socialization processes as major correlates of emotional processing difficulties in this sample. Educational level shows little independent relationship with either construct, suggesting that formal schooling, at least within this university-educated sample, does not substantially reduce cognitive–emotional vulnerabilities that appear to consolidate earlier in development. Together, these results highlight the need for gender-sensitive and culturally adapted cognitive-behavioral approaches that target emotional recognition skills alongside schema-focused work, while remaining attentive to norms around emotional disclosure that differ between male and female participants in this context.

Several limitations require acknowledgment. First, and most fundamentally, the cross-sectional correlational design prohibits any directional inference: the observed associations between cognitive distortions and alexithymia are equally consistent with cognitive distortions contributing to alexithymia, with alexithymia contributing to cognitive distortions, or with both constructs sharing unmeasured developmental antecedents. Although prior longitudinal research indicates that alexithymia is relatively stable across time, the present study cannot speak to temporal ordering, and the language throughout this manuscript has been revised to reflect this constraint. Second, the sample presents a pronounced gender imbalance (71% male, 29% female; *n* = 62 females), which raises two interpretive concerns: the large gender effect sizes observed, including d = 1.23 for total alexithymia, may be partially inflated by this distributional asymmetry, and the moderation and mediation analyses involving gender subgroups may not have been adequately powered, particularly for the female subsample. The gender-related findings should therefore be treated as preliminary and replicated in more balanced samples. Third, the exclusive recruitment of educational and administrative staff from a single university in Riyadh limits representativeness; it is unclear how the findings would generalize to community, clinical, or non-urban adult populations elsewhere in Saudi Arabia or the broader Arab population. Fourth, the absence of any validated measure of psychological distress, mood symptomatology, or general psychological functioning means the clinical significance of the alexithymia levels observed cannot be contextualized against normative expectations; it is not known whether these scores represent elevated levels requiring clinical attention or fall within a normative range for this population. Fifth, the Arabic adaptation of the Cognitive Distortions Scale was conducted by the research team without a published validation study; the adaptation procedure, reliability indices for this sample, and evidence of cultural equivalence should be documented and reported as detailed in Section 3.3.1.

Future research should employ longitudinal designs to address directionality; include validated measures of psychological distress and symptom severity; incorporate the mentalized affectivity framework alongside the TAS−20 to test the conceptual overlap outlined in Section 2.8; recruit from more diverse and representative Arab samples including community, clinical, and non-urban populations; ensure balanced gender subgroup sizes sufficient to support moderation and mediation analyses; and complete and publish a formal validation of any Arabic-language adaptation of the CDS before its use in further research. Cross-cultural comparisons with non-Arab populations would clarify how much of the observed pattern is culturally specific versus transdiagnostically general, and investigations linking culturally shaped cognitive distortions and alexithymia to downstream outcomes such as internet addiction, somatic symptom burden, and help-seeking reticence would strengthen the practical and clinical relevance of the present findings.

## Figures and Tables

**Table 1:** Sample Characteristics (*n* = 214).

Variable	Frequency	Percent	Valid %	Cumulative %
Qualification				
Bachelor’s	112	52.3%	52.3%	52.3%
Master’s/Diploma	63	29.4%	29.4%	81.8%
PhD	39	18.2%	18.2%	100.0%
Gender				
Male	152	71.0%	71.0%	71.0%
Female	62	29.0%	29.0%	100.0%
Occupation				
Teacher	122	57.0%	57.0%	57.0%
Administrative staff	39	18.2%	18.2%	75.2%
Psychologist	53	24.8%	24.8%	100.0%

**Table 2:** Pearson Correlations Between Alexithymia Dimensions and Cognitive Distortions (*n* = 214).

Variable	1	2	3	4	5
1. Cognitive Distortions	—				
2. Difficulty Identifying Feelings	0.29 **	—			
3. Difficulty Describing Feelings	0.19 **	0.62 **	—		
4. Externally Oriented Thinking	0.14 *	0.20 **	0.35 **	—	
5. Total Alexithymia Score	0.28 **	0.85 **	0.84 **	0.60 **	—

Note. * *p* < 0.05. ** *p* < 0.01.

**Table 3:** Gender Differences in Cognitive Distortions and Alexithymia (Independent Samples *t*-test).

Variable	Gender	*n*	M	SD	t	Cohen’s d
Cognitive Distortions	Male	152	65.14	21.35	3.13 **	0.47
	Female	62	55.55	17.60		
Difficulty Identifying Feelings	Male	152	23.84	5.85	6.80 ***	1.02
	Female	62	18.23	4.44		
Difficulty Describing Feelings	Male	152	21.59	3.65	4.41 ***	0.66
	Female	62	18.98	4.50		
Externally Oriented Thinking	Male	152	15.69	3.94	6.50 ***	0.98
	Female	62	11.85	3.84		
Total Alexithymia Score	Male	152	61.12	9.83	8.18 ***	1.23
	Female	62	49.06	9.66		

Note. ** *p* < 0.01. *** *p* < 0.001.

**Table 4:** One-Way ANOVA Results for Differences in Cognitive Distortions and Alexithymia Across Educational Levels.

Variable	Source	SS	df	MS	F	η^2^
Cognitive Distortions	Between Groups	220.66	2	110.33	0.25	0.002
	Within Groups	91,548.63	211	433.88		
	Total	91,769.29	213			
Total Alexithymia Score	Between Groups	533.20	2	266.60	2.15	0.020
	Within Groups	26,132.90	211	123.85		
	Total	26,666.09	213			

Note. η^2^ = eta-squared effect size.

**Table 5:** Hierarchical Regression Predicting Total Alexithymia Score (*n =* 214).

Predictor	B	SE B	β	t	*p*	95% CI	f^2^
Block 1: Demographic Controls							
Gender (male = 1)	13.19	1.47	0.536	8.97	<0.001	[10.29, 16.09]	
Age (centered)	−0.40	0.13	−0.186	−3.08	0.002	[−0.66, −0.14]	
Master’s vs. Bachelor’s	−2.11	1.36	−0.094	−1.55	0.122	[−4.79, 0.57]	
PhD vs. Bachelor’s	−6.73	1.72	−0.233	−3.92	<0.001	[−10.12, −3.34]	
Block 1 R^2^ = 0.335 **				F(4209) = 26.37, *p* < 0.001			0.504
Block 2: + Cognitive Distortions							
Cognitive Distortions	0.077	0.031	0.143	2.46	0.015	[0.015, 0.139]	
Block 2 R^2^ = 0.354, ΔR^2^ = 0.019 *				ΔF(1208) = 6.06, *p* = 0.015			0.029

Note. * *p* < 0.05. ** *p* < 0.001. f^2^ = Cohen’s f^2^ effect size. Incremental f^2^ for distortions = 0.029 (small effect). Block 1 f^2^ = 0.504 (large effect).

**Table 6:** Hierarchical Regression Predicting Alexithymia Subscales: Summary of Block 2 Results (*n* = 214).

Outcome (Block 2)	B	SE B	β	t	*p*	95% CI for B
Difficulty Identifying Feelings (DIF)						
Cognitive Distortions	0.052	0.018	0.177	2.89	0.004	[0.016, 0.087]
Block 2 R^2^ = 0.281, ΔR^2^ = 0.029				ΔF(1208) = 8.34, *p* = 0.004		
Difficulty Describing Feelings (DDF)						
Cognitive Distortions	0.020	0.030	0.043	0.67	0.503	[−0.039, 0.079]
Block 2 R^2^ = 0.157, ΔR^2^ = 0.002				ΔF(1208) = 0.45, *p* = 0.503		
Externally Oriented Thinking (EOT)						
Cognitive Distortions	0.022	0.017	0.079	1.29	0.197	[−0.012, 0.057]
Block 2 R^2^ = 0.231, ΔR^2^ = 0.006				ΔF(1208) = 1.68, *p* = 0.197		

Note. All models control gender, age, and education in Block 1. DIF = Difficulty Identifying Feelings; DDF = Difficulty Describing Feelings; EOT = Externally Oriented Thinking.

## Data Availability

The data presented in this study are available upon request from the corresponding author due to privacy and ethical restrictions related to participant confidentiality.
